# Concerns and Misconceptions About the Australian Government’s COVIDSafe App: Cross-Sectional Survey Study

**DOI:** 10.2196/23081

**Published:** 2020-11-04

**Authors:** Rae Thomas, Zoe A Michaleff, Hannah Greenwood, Eman Abukmail, Paul Glasziou

**Affiliations:** 1 Institute for Evidence-Based Healthcare Bond University Robina Australia

**Keywords:** health, policy, COVID-19, digital tracing app, COVIDSafe

## Abstract

**Background:**

Timely and effective contact tracing is an essential public health measure for curbing the transmission of COVID-19. App-based contact tracing has the potential to optimize the resources of overstretched public health departments. However, its efficiency is dependent on widespread adoption.

**Objective:**

This study aimed to investigate the uptake of the Australian Government’s COVIDSafe app among Australians and examine the reasons why some Australians have not downloaded the app.

**Methods:**

An online national survey, with representative quotas for age and gender, was conducted between May 8 and May 11, 2020. Participants were excluded if they were a health care professional or had been tested for COVID-19.

**Results:**

Of the 1802 potential participants contacted, 289 (16.0%) were excluded prior to completing the survey, 13 (0.7%) declined, and 1500 (83.2%) participated in the survey. Of the 1500 survey participants, 37.3% (n=560) had downloaded the COVIDSafe app, 18.7% (n=280) intended to do so, 27.7% (n=416) refused to do so, and 16.3% (n=244) were undecided. Equally proportioned reasons for not downloading the app included privacy (165/660, 25.0%) and technical concerns (159/660, 24.1%). Other reasons included the belief that social distancing was sufficient and the app was unnecessary (111/660, 16.8%), distrust in the government (73/660, 11.1%), and other miscellaneous responses (eg, apathy and following the decisions of others) (73/660, 11.1%). In addition, knowledge about COVIDSafe varied among participants, as some were confused about its purpose and capabilities.

**Conclusions:**

For the COVIDSafe app to be accepted by the public and used correctly, public health messages need to address the concerns of citizens, specifically privacy, data storage, and technical capabilities. Understanding the specific barriers preventing the uptake of contact tracing apps provides the opportunity to design targeted communication strategies aimed at strengthening public health initiatives, such as downloading and correctly using contact tracing apps.

## Introduction

COVID-19 is a viral disease caused by a newly discovered strain of coronaviruses. People affected by the disease commonly present with fever, cough, and shortness of breath. This disease can also cause death, with varying rates observed in different countries. In Australia, the first case of COVID-19 was confirmed in late January 2020, with the first wave occurring between March and May, 2020. In the absence of a vaccine, nondrug interventions for preventing COVID-19 and any other future infectious outbreaks are critical [1,2]. The public has been asked to practice preventive behaviors, such as hand hygiene, physical distancing, quarantining, and getting tested when sick. These behaviors are being promoted by national and international public health organizations through population-based communication strategies. Alongside individually practiced prevention strategies are population-based strategies such as contact tracing, which is critical for preventing and slowing the spread of disease.

To improve public health contact tracing and the speed at which it occurs, several countries have introduced app-based contact tracing. Contact tracing apps vary in design, from reporting symptoms to public health authorities [3] to allowing access to phone data after testing positive for COVID-19 [4]. They also vary in whether the data are centralized [5]. Furthermore, contact tracing apps in current use have had varying degrees of success [3,6]. Since the Australian Government launched the COVIDSafe app in late April 2020 [4], over 6 million Australians (almost 25%) have downloaded the app. However, worldwide concerns have been raised about the privacy and ethics of this digital approach [7], which may hamper app downloads and decrease app effectiveness. This has been reflected in the Australian uptake of the COVIDSafe app; following its initial release, downloads have progressively decreased. Currently, Australian downloads are short of the 40% proposed target for the app to be effective, and this has not been anticipated to change without further government intervention to increase uptake.

App-based contact tracing requires public cooperation. Individuals are required to install the app, keep Bluetooth functions on, have the app activated or open on their phones, and carry their phones with them when outside of their home. This sounds simple, but when considered from a behavior change perspective, these behaviors are complex and need to be performed together to optimize contact tracing functionality [8]. To identify behavior change techniques for improving the uptake of app-based contact tracing, we first need to understand people’s reasons for not downloading the app. In this study, we aimed to investigate the uptake of the Australian Government’s COVIDSafe app among Australians, identify Australians’ understanding of the purpose and capabilities of the app, and explore the reasons why some Australians chose not to download the app.

## Methods

Participants were recruited for a national, cross-sectional, online survey by the panel provider, Dynata. The use of a panel provider for online research provides confidence in attaining a representative sample of the required size and allows for quick completion of time-sensitive projects. The panel provider adheres to our quotas for age, gender, and state/territory of residence, ensuring that our sample is representative of the broader population. Through Dynata, participants received points for completing the survey, which may be used for gift vouchers, donations, or cash redemption. Our sample was representative of all Australian states and territories and met our quotas for age and gender. Participants were included in our study if aged ≥18 years. Participants were excluded if they had, or thought they had, COVID-19. They were also excluded if they were health care professionals, as this group may have systematic differences in knowledge of COVID-19 compared to the general Australian population.

Prior to screening, potential participants read detailed study information, including eligibility criteria, what the study involved, and privacy and confidentiality rights. Participants were informed that commencing the survey indicated their informed consent to participate in this study. Ethics approval was obtained from the Bond University Human Research Ethics Committee (#RT03008).

All participants were asked whether they had downloaded, or intended to download, the COVIDSafe app. If they responded “unsure” or “no intention to download,” they were asked to provide a reason for their response. We qualitatively coded the reasons for inaction and uncertainty and conducted a thematic content analysis of open-ended responses. Uninformative responses, such as “not sure,” were not coded. If multiple concerns were mentioned, only the first response was coded. The code frame was initially developed by RT, and then discussed and refined by the other authors. Afterward, 1 author (RT) completed the qualitative analysis of all responses. Participants then rated their strength of agreement for 6 statements related to the app’s purpose and capabilities using a 5-point Likert scale (1=strongly disagree to 5=strongly agree; option for “don’t know” response was available). The survey items and response scale are available in Multimedia Appendix 1.

## Results

Of the 1802 potential participants contacted, 289 (16.0%) were screened as ineligible prior to completing the survey and were excluded, 13 (0.7%) declined, and 1500 (83.2%) participated in the survey. There was representation across all adult age groups and sexes (50.0% male), and education levels were distributed evenly (high school and technical and further education qualification or lower: 735/1500, 49.0%; tertiary qualification: 765/1500, 51.0%) (Table 1).

Of the 1500 survey participants, 37.3% (560/1500) said they downloaded the COVIDSafe app, 18.7% (280/1500) had intended to, 27.7% (416/1500) refused, and 16.3% (244/1500) were undecided. Of the 660 who refused or were undecided, 25.0% (n=165) cited privacy concerns as their primary reason. For example, many distrusted the security of the app; some participants believed that the COVIDSafe app was not safe and that it could be hacked, resulting in their information being used without their authority. Another 24.1% (159/660) cited technical problems, such as phones being too old or limitations in data consumption and storage space. Other reasons for being undecided or refusing to download the app included the belief that social distancing was sufficient and the app was unnecessary, distrust in the government, questioning the app’s effectiveness, wanting to explore more information before deciding, and other miscellaneous responses, such as apathy and following the decisions of others (Table 2).

**Table 1:** Participants’ characteristics (N=1500).

Characteristics		Values, n (%)
Female		750 (50.0)
**Age (years)**		
	18-24	171 (11.4)
	25-34	264 (17.6)
	35-4	239 (15.9)
	45-54	223 (14.9)
	55-64	222 (14.8)
	65-74	227 (15.1)
	≥75	154 (10.3)
**Education**		
	High school graduate or lower	459 (30.6)
	Trade certificate (I-IV)	276 (18.4)
	Tertiary	765 (51.0)
**Australian states and territories**		
	Queensland	302 (20.1)
	New South Wales	471 (31.4)
	Australian Capital Territory	29 (1.9)
	Northern Territory	9 (0.6)
	Western Australia	160 (10.7)
	Victoria	382 (25.5)
	Tasmania	34 (2.3)
	South Australia	113 (7.5)
**Aboriginal or Torres Strait Islander**		
	Yes	17 (1.1)
	No	1471 (98.1)
	Prefer not to say	12 (0.8)
Born in Australia		1049 (69.9)

**Table 2:** Reasons for not downloading the COVIDSafe app (N=660).

Reasons for not downloading	Values, n (%)
Privacy concerns	165 (25.0)
Technical problems	159 (24.1)
App is unnecessary	111 (16.8)
Distrust in the government	73 (11.1)
Questioning effectiveness of app	46 (7.0)
Need more information before deciding	33 (5.0)
Uncoded miscellaneous reasons	73 (11.1)

**Figure 1:**
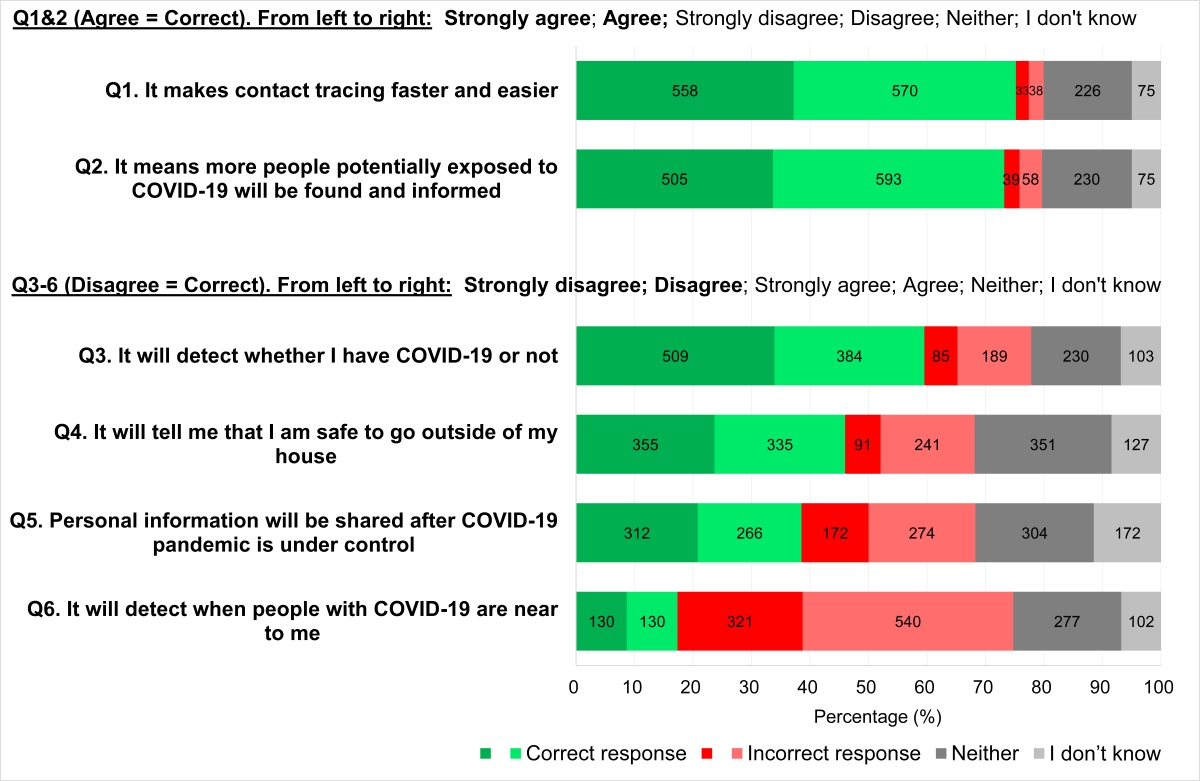
Participants’ ratings for the suggested purposes and capabilities of the COVIDSafe app (N=1500).

With respect to the app’s intended purpose and capabilities, almost 75% of participants correctly agreed that the app would make contact tracing faster and easier (strongly agree: 558/1500, 37.2%; agree: 570/1500, 38.0%), and almost 72% correctly agreed that more people who were potentially exposed to COVID-19 would be found and informed (strongly agree: 505/1500, 33.7%; agree 593/1500, 39.5%) (Figure 1). In contrast, almost 50% of participants incorrectly thought that their personal information would be shared after the pandemic (strongly agree: 172/1500, 11.5%; agree: 274/1500, 18.3%; neither: 304/1500, 20.3%), and almost 72% incorrectly thought that the app would detect when people with COVID-19 were near them (strongly agree: 321/1500, 21.4%; agree: 540/1500, 36.0%; neither: 227/1500, 15.1%) (Figure 1). Interestingly, participants were divided in knowing whether the app would inform them that it was safe to leave their house (Figure 1).

In Table 3, we descriptively report the differences in intentions to download the COVIDSafe app between age groups. We did not perform statistical analyses on these subgroups. However, there appears to be little difference between age groups in terms of the number of app downloads and the number of people who intended to download the app. For example, in the youngest age group, 60.8% (104/171) of our sample had already downloaded, or intended to download, the app, and 58.4% (90/154) of participants in the oldest age group had done, or intended to do, the same (Table 3). This pattern was also observed for those who decided to not download the app or were undecided.

**Table 3:** Age groups and intentions to download the COVIDSafe app.

Age group (years)	Downloaded, n (%)	Intend to download, n (%)	Refused to download, n (%)	Unsure, n (%)	Total
18-24	52 (30.4)	52 (30.4)	50 (29.2)	17 (9.9)	171
25-34	94 (35.6)	69 (26.1)	57 (21.6)	44 (16.7)	264
35-44	91 (38.1)	39 (16.3)	67 (28.0)	42 (17.6)	239
45-54	82 (36.8)	33 (14.8)	70 (31.4)	38 (17.0)	223
55-64	82 (36.9)	31 (14.0)	65 (29.3)	44 (19.8)	222
65-74	97 (42.7)	28 (12.3)	65 (28.6)	37 (16.3)	227
≥75	62 (40.3)	28 (18.2)	42 (27.3)	22 (14.3)	154
Total^a^	560 (37.3)	280 (18.7)	416 (27.7)	244 (16.3)	1500

^a^Total n for each download behavior and % of total sample.

## Discussion

Timely and effective contact tracing is an essential public health measure for curbing the transmission of COVID-19. Contact tracing apps are controversial in their design and level of effectiveness [3,5,6], but they might have the potential to prevent widespread community transmission and optimize the resources of overstretched public health organizations [9]. An important driver for their efficiency is widespread public adoption [9]. Our study aimed to examine Australian participants’ understanding of the Australian Government’s COVIDSafe app and explore the reasons why some Australians chose not to download the app. Primarily, we found that while most people correctly understood the intended purpose and capabilities of the app, there was some crucial misunderstandings about the contact tracing limits of the software (ie, whether the app can detect when a person is close to someone with COVID-19 and whether the app can let people know when it is safe to leave home). Among those who were undecided or refused to download the app, the main reasons for hesitation centered around privacy and technological barriers, which are key concerns that need to be addressed if uptake of the app is to increase.

In total, 37.3% (560/1500) of our sample said they had downloaded the app and 18.7% (280/1500) intended to. This proportion is concordant with another Australian survey with a smaller online sample (N=439), in which 44.0% of participants reported downloading the COVIDSafe app [10]. However, based on other surveys in which participants were asked whether they intended to download hypothetical apps, the acceptability of contact tracing apps is higher in other countries. For example, in a recent online survey from Ireland (N>8000) [11], when asked about downloading a contact tracing app that was not yet available, 58% of participants said they would download it and 25% said they probably would [8]. Additionally, in another online survey with almost 6000 participants from 5 countries (ie, the United Kingdom, Germany, Italy, France, and the United States), 75% of participants said they definitely or probably would install a contact tracing app [12]. It would be interesting to see whether people will actually follow through with their intentions to download a contact tracing app.

With regard to open-ended text responses, 25% of participants in our study who did not download the COVIDSafe app were concerned about privacy. This is lower than the 31% in the smaller Australian study [10] and the 41% in the Irish study [11] who believed privacy was a problem. The differences in these percentages may be due to the free-text responses available in our survey, as the other studies used a list of options for participants’ responses. Additionally, compared to the 11.1% of participants in our survey who did not download the app because they distrusted the government, there was more distrust in postpandemic government surveillance with Irish participants (33%) [11] and participants in the cross-country survey (42%) [12]. When considering communication strategies for improving contact tracing app downloads and use, better communication approaches are needed to put the public’s concerns about privacy and the government at ease.

Our study also reveals that there are missed communication opportunities for correcting erroneous beliefs about the capabilities of the COVIDSafe app. Over half of our participants (810/1500, 54.0%) thought the COVIDSafe app would or might tell them when it was safe to leave the house, and 40.5% (607/1500) thought it would or might tell them whether they had COVID-19 (Figure 1). Addressing these perceptions and issues about the capabilities of the app with public messaging is important for achieving sufficient uptake of contact tracing apps.

Based on reviewer feedback and the fact that older adults are disproportionally affected by COVID-19, we performed a posthoc analysis to descriptively examine the uptake of the app by age. App downloads appeared to increase with age, with the 65-74-year and ≥75-year age groups having the highest proportion of downloads, and this trend may reflect older adults’ vulnerability to COVID-19. However, when the number of people who downloaded or intended to download the app were combined, the differences between age groups were much smaller. Almost one-third (416/1500, 27.7%) of participants, regardless of age, chose not to download the app.

To our knowledge, this study is the first to qualitatively analyze open-ended text responses to barriers for downloading a contact tracing app. Compared to having participants select a response from a list of predefined options, our approach decreases potential researcher biases and strengthens the ability to inform communication techniques for improving app uptake. To further minimize bias, we deliberately recruited a sample with representation from all Australian states and territories and quotas for age and gender. We believe this strategy improved the generalizability of our findings to the broader Australian population. However, we did not assess cultural and linguistic diversity. Therefore, the generalizability of our findings is limited to Australians, and our results may not reflect the perceptions of individuals whose primary language is not English.

Although the level of effectiveness of contact tracing is still unclear [3,6], apps have been used in various countries to help with both contact tracing [4] and medical management of COVID-19 cases [13]. Their utility comes from being used as an adjunct contact tracing strategy alongside public health staff, particularly when community transmissions are high. For apps to be accepted by the public and used correctly, we need to better communicate concerns about privacy, data storage, and technical capabilities. The lessons learned during the COVID-19 pandemic will be invaluable for inevitable future infectious outbreaks.

## Appendix

Multimedia Appendix 1 Table detailing the COVIDSafe survey items and scores.
