# Supplementary material for: Concerns and Misconceptions About the Australian Government’s COVIDSafe App: Cross-Sectional Survey Study
Source: JMIR Public Health Surveill. 2020 Nov 4;6(4):e23081. doi: 10.2196/23081 (PMC7644267; doi:10.2196/23081)
Supplement: Multimedia Appendix 1 [file publichealth_v6i4e23081_app1.DOC]

**COVID Safe Download Status**

The Australian Government has recently asked us to download the COVID-19 tracing app called COVIDSafe. Which of the following applies to you?

- I have already downloaded the COVIDSafe app
- I intend to download the COVIDSafe app but haven’t yet
- I do not intend to download the COVIDSafe app
- I am not sure whether I will download the COVIDSafe app

**Reasons for not downloading the app? (Open ended, asked of those who do not intend to or are unsure whether to download the app)**

Why do you think you would not, or might not, download the app?

**Agreement with statements about COVIDSafe app (randomly ordered)**

How much do you agree or disagree with the following statements about the COVIDSafe app?

- It makes contact tracing faster and easier 1 – 5 strongly disagree to strongly agree and a ‘don’t know’ option
- It means more people potentially exposed to COVID-19 will be found and informed 1 – 5 strongly disagree to strongly agree and a ‘don’t know’ option
- It makes it easier to track contacts across state borders 1 – 5 strongly disagree to strongly agree and a ‘don’t know’ option
- Personal information will be shared after COVID-19 pandemic is under control 1 – 5 strongly disagree to strongly agree and a ‘don’t know’ option
- It will tell me that I am safe to go outside of my house 1 – 5 strongly disagree to strongly agree and a ‘don’t know’ option
- It will detect when people with COVID-19 are near to me 1 – 5 strongly disagree to strongly agree and a ‘don’t know’ option
- It will detect whether I have COVID-19 or not 1 – 5 strongly disagree to strongly agree and a ‘don’t know’ option
